# Value of Robotics: Comparison of Three Different High-Intensity Training Programs for Rehabilitation After Stroke

**DOI:** 10.3390/s25247667

**Published:** 2025-12-18

**Authors:** Nándor Prontvai, Szilvia Kóra, Blanka Törő, Barbara Kopácsi, Petra Kós, Tamás Haidegger, György Wersényi, Péter Prukner, István Drotár, József Tollár

**Affiliations:** 1 Somogy County Mór Kaposi Teaching Hospital, H-7400 Kaposvár, Hungary; prontvai.nandor@kmmk.hu (N.P.); kos.petra.klaudia@kmmk.hu (P.K.); tollar.jozsef@kmmk.hu (J.T.); 2 Doctoral School of Health Sciences, Faculty of Health Sciences, University of Pécs, H-7621 Pécs, Hungary; 3 University Research and Innovation Center (EKIK), Óbuda University, H-1034 Budapest, Hungary; 4 School of Computing, Queen’s University, Kingston, ON K7L 3N6, Canada; 5 Department of Telecommunications, Széchenyi István University, H-9026 Győr, Hungary; wersenyi@sze.hu; 6 Digital Development Center, Széchenyi István University, H-9026 Győr, Hungary

**Keywords:** stroke, rehabilitation, high intensity, agility, robotic, augmented reality, virtual reality, treadmill training, exergaming, gait

## Abstract

Strokes are one of the leading causes of adult disability. There are a wide range of therapies available in stroke care for people with stroke, but there can be wide variations in the effectiveness of these therapies, so it is essential to review and compare them from time to time. In our study, we measured and compared the effectiveness of three high-intensity therapies: an agility training program without technological tools, a virtual reality exergaming training program with a low-cost device, and a high-cost robotic training program using augmented and virtual reality. All three therapies helped to improve the patients’ functional abilities, balance, and gait. On average, endurance increased by 104–177%, balance scores by 36–53%, and gait speed by 5–10% depending on the intervention. Robotic therapy and exergaming facilitate greater improvements in walking speed, step length, and balance-related gait metrics. These findings have profound implications for stroke rehabilitation, advocating for the prioritization of robotic and exergaming interventions over conventional functional therapies, like agility training. Given the limited sample size, the results should be interpreted as preliminary, highlighting the need for further studies with larger cohorts.

## 1. Introduction

Stroke remains one of the most significant public health challenges in Europe, being the second leading cause of death and a major contributor to long-term adult disability. Each year, around 1.1 million individuals suffer a stroke in the European Union, resulting in approximately 440,000 deaths. The economic burden is similarly profound. In 2017, the total cost of stroke across Europe was estimated at EUR 45 billion, covering both direct medical care and indirect costs such as long-term support and lost productivity. These figures are projected to grow rapidly as life expectancy increases and age-related vascular conditions become more prevalent in the population [1].

Geographically, Central and Eastern European countries carry a disproportionately high share of stroke-related morbidity and mortality. Although many Eastern European nations have made progress in aligning their care systems with Western European standards, structural inequalities remain, especially in the availability of post-acute rehabilitation services and advanced therapeutic technologies [2]. In many regions, stroke survivors receive only limited access to modern neurorehabilitation techniques, which may delay or limit functional recovery. This situation is particularly acute in low- and middle-income countries, where constraints on infrastructure and workforce often prevent access to comprehensive acute care. In such settings, post-stroke rehabilitation becomes not only a critical phase of recovery but potentially the most impactful intervention available to preserve and restore function.

Contemporary stroke rehabilitation offers a wide range of therapeutic approaches to address motor deficits, impaired mobility, and loss of functional independence. Among these interventions, conventional physical therapy techniques—such as balance training, strength conditioning, and task-specific motor practice—are widely used and remain the foundation of care. However, emerging technologies, including virtual reality (VR)-based exergaming, functional electrical stimulation, and robotic-assisted therapy, have shown promise in accelerating recovery by promoting repetitive high-intensity goal-directed movement patterns that facilitate neural reorganization [3].

A growing body of literature has confirmed that high-intensity training is a key driver of neuroplasticity, the brain’s capacity to adapt and reorganize in response to injury. Intensive rehabilitation programs have been associated with improved motor and functional outcomes not only in stroke but also in other neurological conditions, such as Parkinson’s disease and multiple sclerosis [4,5,6,7]. In the context of stroke, high-intensity interventions appear to support sensorimotor reintegration, balance control, and gait symmetry, especially when delivered early in the recovery trajectory.

Technologically enhanced rehabilitation platforms, such as robotic gait trainers or immersive VR environments, can standardize and amplify the effects of high-intensity therapy by enabling precise, repetitive, and quantifiable movement tasks. These devices can also capture real-time feedback, which has been associated with increased patient engagement and motivation. Several studies have demonstrated that robotic-assisted therapy, in particular, can improve walking endurance, balance, and lower-limb coordination [8,9,10,11,12,13,14,15]. However, many of these studies have evaluated a single modality in isolation, often under pilot conditions, with relatively small sample sizes and variable protocols. As a result, few robust comparative studies exist that examine how these technological approaches perform against each other and against well-designed conventional programs.

Recent investigations have provided valuable insights into this area. Robotic-assisted rehabilitation has been shown to improve not only clinical motor outcomes but also neurophysiological parameters such as cortical excitability, suggesting the induction of use-dependent plasticity in stroke-affected brain regions [16]. Platforms incorporating exergaming and motion tracking have been linked to enhanced user adherence and increased training volume, both of which are key to maximizing functional gains [17]. International guidelines have begun to include robotic gait training as a recommended intervention; however, clear protocols concerning frequency, intensity, and patient selection are still lacking [15]. Some research has also explored hybrid approaches, such as combining mirror therapy with robotics, which appear to reinforce self-efficacy and participation in recovery [18]. Technologies such as brain–computer interfaces and multimodal virtual environments are also gaining recognition, especially for patients in the chronic phase of stroke who have limited benefits from conventional interventions [19].

Despite this progress, important questions remain unanswered. There is a need for direct comparison of these modalities using objective biomechanical and clinical outcome measures under real-world conditions. It is particularly important to understand how robotic therapy, screen-based interactive exergaming, and high-intensity conventional therapy differ in their ability to restore walking ability, improve balance control, and support independent movement in daily life.

The main objective of this study is to conduct a head-to-head comparison of three different high-intensity rehabilitation approaches—robotic-assisted therapy, screen-based interactive exergaming, and conventional agility-based physiotherapy—in individuals recovering from subacute ischemic stroke. The study aims to determine which of these interventions produces the greatest improvements in functional walking ability, gait parameters (such as walking speed, step length, and cadence), and postural stability. All three interventions are delivered at comparable intensity levels and within the same timeframe, ensuring consistency in dosage and allowing meaningful comparisons to be drawn. Beyond the primary clinical goal, the study also examines secondary dimensions of effectiveness that are increasingly relevant in rehabilitation practice. These include patients’ perceived effort and engagement during therapy, the overall feasibility of implementing each modality in hospital or outpatient settings, and the extent to which different interventions support consistent attendance and task adherence. Particular attention is paid to how the structure and feedback mechanisms of each therapeutic platform influence patient motivation and rehabilitation performance. The study also explores whether the observed clinical gains correlate with measurable differences in movement quality, variability, or exercise-induced fatigue, as captured by instrumented gait analysis systems.

Recent meta-analyses and systematic reviews highlight the increasing clinical relevance of robotic and VR-assisted rehabilitation approaches, underscoring the need for comparative studies under real-world conditions [20,21,22].

The remainder of this paper is organized as follows: Section 2 presents the materials and methods, including details of the study design, participant characteristics, outcome measures, intervention protocols, and statistical analyses. Section 3 reports the main results of the interventions. Section 4 discusses the findings in the context of the existing literature on post-stroke neurorehabilitation and neural plasticity mechanisms. Finally, Section 5 summarizes the main conclusions, highlighting the clinical implications and directions for future research.

## 2. Materials and Methods

### 2.1. Study Design

The present study is a quantitative randomized blinded pre-post prospective clinical trial for which data were collected at the Neurological Rehabilitation Unit of the Neurological Department of the Somogy County Mór Kaposi Teaching Hospital. The aim of the present study is to determine and compare the effects of high-intensity robotic training (ROB), high-intensity agility training (AG), and high-intensity exergaming (EX) on clinical symptoms, function, and mobility for people with subacute ischemic stroke. Screen-based interactive exergaming was delivered through a sensor-driven movement platform using visual feedback via large display screens (not immersive VR headsets), designed to promote task-oriented functional movement through engaging game-like tasks.

### 2.2. Participants

The study was conducted among patients with subacute ischemic stroke. Participants were identified from the hospital database and screened for inclusion in the study. A total of 42 subacute ischemic stroke survivors were identified and examined, of whom 9 were excluded (did not meet inclusion criteria: *n* = 4; refused participation: *n* = 5), giving a total sample size of 33. Detailed baseline characteristics of the participants, including physical parameters, sex distribution, affected brain area, comorbidities, and destructive habits, are summarized in Table 1.

Participants were randomly assigned to one of the three intervention groups (ROB, AG, and EX) using a computer-generated random sequence created in Microsoft Excel. To ensure allocation concealment, the randomization list was prepared and held by an independent researcher who was not involved in participant recruitment, assessment, or intervention delivery. Group assignments were placed in sequentially numbered opaque sealed envelopes, which were opened only after baseline assessment. This procedure minimized potential selection bias and preserved the integrity of the randomization process. To ensure comparability, baseline demographic and clinical characteristics (such as age, sex, stroke side, modified Rankin Scale (mRS), and Functional Independence Measure (FIM) scores) were statistically analyzed before the intervention. No significant differences were found among the groups, confirming baseline equivalence.

Inclusion criteria for the study were as follows: first ischemic stroke diagnosed by a neurologist on computed tomography (CT) or magnetic resonance (MR) imaging; time since stroke between 2 and 4 weeks; neurological examination revealed mobility and postural limitation; mRS score was 2 or higher. The exclusion criteria are history of multiple strokes; systolic blood pressure less than 120 or higher than 160 mmHg; orthostatic hypotension; carotid artery stenosis; severe heart disease; hemophilia; traumatic brain injury; seizure disorder; untreated diabetes; abnormal electroencephalography; Mini-Mental State Examination (MMSE) score < 22; abnormal blood panel; use of sedatives; irregular medication; severe aphasia (Western Aphasia Battery ≤ 25); severe visual or hearing impairment; severe sensory dysfunction; severe orthopedic problem; other neurological condition affecting motor function; alcoholism; drug use; smoking after diagnosis of stroke; inability to walk at least 100 m with or without an assistive device in 6 min; inability to understand verbal instructions or signals on a television screen; current participation in an individual or group exercise program outside of standard physiotherapy.

### 2.3. Patient Allocation

The selected participants were randomly allocated in equal proportions to one of the groups: ROB (*n* = 11) receiving robotic therapy, AG (*n* = 11) receiving agility training, and EX (n = 11) receiving exergaming. Prior to the study, all patients had received standard physiotherapy care funded by social security. Participants in all three groups suspended this care and only participated in the intervention training programs.

### 2.4. Outcomes

Outcomes were measured before and after the intervention. The measurements were taken by the same person each time, and the grouping of participants was hidden from him/her. The testing method was standardized for all participants at each measurement session. Pre- and post-tests were conducted within 1 week of the intervention. Measurements were conducted in the dimensions of functional ability, balance, and gait. The measures and tests listed below are excellent tools for assessing the condition of stroke patients as they cover a wide range of stroke-affected functions and provide objective reliable data for planning and monitoring the rehabilitation process. The combined use of these tools allows a comprehensive multidimensional assessment of stroke patients, taking into account physical function, mobility, and balance. This detailed information is essential for designing personalized rehabilitation programs, monitoring progression, and evaluating the effectiveness of treatment. In addition, patient rate of perceived exertion (RPE) was recorded after exercise during each training session. Peak heart rate during exercise was also recorded.

#### 2.4.1. Functional Abilities

The mRS indicates the degree of independence in daily activities and the severity of disability. The mRS is a reliable and validated measure that is sensitive to changes over time. The method for assessing mRs is a guided interview. The assessment involves asking the patient about activities of daily living, including outdoor activities. The assessment should take into account any neurological deficits (e.g., aphasia and intellectual deficits) detected during the assessment. Finally, aspects of the patient’s physical state, mental performance, and speech should be combined when selecting the mRS score. The scoring is divided as follows: 0—No symptoms; 1—No significant disability. Able to carry out activities of daily living despite some symptoms; 2—Mild disability. Able to look after himself without assistance but not able to carry out all previous activities; 3—Moderate disability. Needs some assistance with activities of daily living but can walk without assistance; 4—Moderately severe disability. Unassisted, unable to meet his/her own physical needs, unable to walk without assistance; 5—Severe disability. Bedridden, incontinent, requires constant nursing care; 6—Dead [23].

We used Barthel Index (BI) to measure functional ability, which sensitively measures the degree of independence and changes in this during various activities of daily living (eating, transfers to wheelchair, personal toilet, toilet use, bathing, walking on flat ground, up and down stairs, dressing, and defecation and urination) in chronic disabling conditions, especially in rehabilitation settings. The scoring method takes into account whether the person being assessed receives assistance in performing each task. The scores for each item are summed, and the resulting total score represents the BI score on a scale of 0–100; the higher the score, the greater the autonomy [24].

The Schwab and England Scale (SE-ADL) assesses the abilities of people living with a disease in relation to various life activities on a scale of 0–100%. The percentage score is determined by the examiner based on a description of the patient’s abilities and difficulties in routine life activities: 100%: Completely independent. Able to do all chores without slowness, difficulty or impairment. Essentially normal. Unaware of any difficulty.; 90%: Completely independent. Able to do all chores with some degree of slowness, difficulty and impairment. Might take twice as long. Beginning to be aware of difficulty.; 80%: Completely independent in most chores. Takes twice as long. Conscious of difficulty and slowness.; 70%: Not completely independent. More difficulty with some chores. Three to four times as long in some. Must spend a large part of the day with chores.; 60%: Some dependency. Can do most chores, but exceedingly slowly and with much effort. Errors; some impossible.; 50%: More dependent. Help with half, slower, etc. Difficulty with everything. 40%: Very dependent. Can assist with all chores, but few alone. 30%: With effort, now and then does a few chores alone or begins alone. Much help needed. 20%: Nothing alone. Can be a slight help with some chores. Severe invalid. 10%: Totally dependent, helpless. Complete invalid. 0%: Vegetative functions such as swallowing, bladder and bowel functions are not functioning. Bedridden [25].

The Functional Independence Measure (FIM) was used to measure functional independence. The FIM is an 18-item scale consisting of motor and cognitive subscales. The assessment includes tests of self-care, bladder control, transfers, locomotion, communication, and cognitive functions. Each item is rated on a scale of 1 to 7, with 1 indicating maximum need for assistance and 7 indicating complete independence. Accordingly, the total scale scores range from 18 to 126, where 18 indicates total dependence and 126 indicates total independence. Each subscale can be scored separately; in the present study, the FIM motor subscale was also scored independently. In this case, the full scale can range from 13 to 91, where the higher the value the greater the motor independence [26].

#### 2.4.2. Balance and Postural Stability

The Berg Balance Scale (BBS) was used to measure static and dynamic balance. The BBS can objectively measure the subject’s ability to balance safely during a series of predetermined tasks. It is a 14-item task, with each item scored on a five-point scale from 0 to 4, where 0 indicates the lowest level of function and 4 the highest. The items are as follows: standing up from a sitting position; standing without assistance and without grasping; sitting without supporting the back with the feet resting on the floor; sitting down from a standing position; transfer movement from one chair to another; standing with eyes closed without assistance and without grasping; standing with legs together without assistance and without grasping; reaching forward with outstretched arm in standing position; picking up an object from the floor in standing position; looking back over right and left shoulder in standing position; 360 degree turn in both directions; alternate foot steps up and down stairs or stool; standing without assistance or support with one foot in front of the other; standing on one foot. The maximum score is 56, indicating perfect functional balance. A score below 45 indicates an increased risk of falling, and, the lower the score, the higher the risk of falling [27].

To assess functional balance, we used the Balance Evaluation Systems Test (BESTest), a 36-item test that evaluates 6 different balance systems: biomechanical constraints, stability limits/verticality, anticipatory responses, postural responses, sensory orientation, and stability in gait. Patients performed the testing method in flat-soled shoes. The instrument consists of 27 tasks and assesses 36 items in total. Each item was scored between 0 and 3, where 0 represents the worst performance and 3 represents the best performance. A maximum total of 108 points can be achieved in the test, the percentage of which provides the final score. The higher the score, the better the balance is assumed [28].

Fear of falling was measured using the Falls Efficacy Scale (FES). This is a 10-item questionnaire that asks participants to indicate on a scale of 1 to 10 how confident they are in performing various everyday tasks without fear of falling. The dimensions of the questionnaire are as follows: bathing/showering; taking objects out of the cupboard; walking every day; preparing meals; going to bed/getting up; opening the door/answering the phone; getting up from/getting down from a chair; getting dressed/dressed; personal hygiene; getting down/up from the toilet. A total of 100 points can be obtained by adding up each dimension, with a summed score greater than 70 indicating that the respondent is clearly afraid of falling [29].

During the postural examination, the presence and extent of postural instability were monitored using a posturograph (ProKin 252N, TecnoBody, Dalmine, Italy). During the test, the subjects were asked to stand on the test apparatus for 20 s in 4 different increasingly difficult postures: 1—wide stance-eye open (WEO); 2—wide stance-eye closed (WEC); 3—narrow stance-eye open (NEO); 4—narrow stance-eye closed (NEC). During the test, the subjects stood barefoot on the test apparatus and were not exposed to any external stimuli during the test. The results are provided by the 3-dimensional path of the body’s center of pressure (COP) (COP Sway) expressed in millimeters and the area covered by at least 95% of the COP path (COP Area) expressed in square centimeters; the smaller the COP Sway, the lower the postural instability. In addition, the device can be used to measure the speed of movement of the COP (COP Velocity), which also implies a lower degree of holding instability the slower it is [30,31,32,33].

#### 2.4.3. Gait Assessments

To measure endurance, we used a 6-min walk test (6mWT). During the test, the subject walks back and forth along a pre-designated flat and straight 50 m long trail. The walk starts when the timer is started at the tester’s command; the pace of the walk is set by the subject, with no interference from the tester. If the subject needs to stop for a rest during the walk, the timing is not stopped. The subject will continue walking for 6 min and will stop only at the direction of the examiner after the 6 min have elapsed. The result is the distance covered in 6 min, expressed in meters. If the subject is required to stop and sit down within the 6 min, the test result is the distance covered. To ensure accurate measurement, the 50 m long marked track is marked with thick tape every 10 m, and the distance between thick tapes is marked with thinner tape every 2 m [34].

A 10-m walk test (10mWT) was used to measure walking speed. To perform the test, a 10 m long flat, level area was designated. The 10 m section was marked at 0, 2, 8 and 10 m with thick adhesive tape. The subject started walking at the 0 m mark on the instructions of the examiner. As soon as he reached the 2 m mark, the examiner started the timer and stopped it the moment he reached the 8 m mark, thus testing his walking speed over a 6 m stretch. The speed is given by the distance (6 m) divided by the time measured (in seconds to two decimal places) in m/s [35].

**Figure 1:**
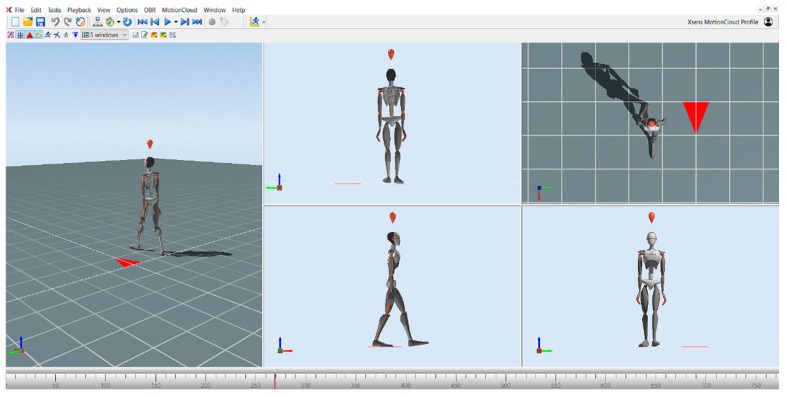
MVN Analyze motion analysis software during gait analysis recording.

Gait parameters (speed, cadence, step length, step width, temporal parameters of step, swing phase, stance phase, and double support phase) were also measured using the MVN Awinda motion capture system developed by Xsens (Movella, El Segundo, CA, USA) and the MVN Analyze (version 2025.0.1.) motion analysis software (Figure 1). The MVN Awinda motion capture system consists of a total of 17 inertial measurement units (IMUs), which include accelerometers, gyroscopes, and magnetometers. The sensors are placed at different predefined anatomical points on the body. These points are midfoot, medial surface of the proximal tibia, lateral surface of the proximal femur, sacrum, sternum, shoulder blades, lateral surface of the proximal humerus, distal forearm, back of the hand, and head. This layout accurately represents the body’s motion in three-dimensional (3D) space and provides a high level of temporal and spatial accuracy during motion capture. After the sensors have been successfully attached, the subject’s anthropometric data must be provided to the software prior to the calibration process. In our test, the body height and the inside foot height were entered, from which the software calculates the limb lengths. This data is important for the software to accurately simulate the anatomy and mechanical behavior of the body. The calibration process consists of two phases: a static resting position, followed by a short predefined movement. There are two types of static calibration: the T-pose and the N-pose. In the T-pose, the subject keeps the arms extended horizontally and the legs parallel. In the N-pose, the subject remains in a natural standing position, allowing the system to calibrate the relative position of the sensors directly from the resting position. The second stage of the calibration was the dynamic calibration, during which we asked the patients to turn around and walk for a short time. The walk was also performed for a few seconds in order to allow the system to calibrate the relative position of the sensors and the movement patterns of the different segments of the body. After the walk, the initial position was resumed and held for approximately 1 min. After successful calibration, the gait analysis was started. During gait analysis, a 15 m section was pre-measured, which each patient had to complete without an assistive device. Before recording the walk, the subjects were instructed to walk at a comfortable walking speed at their own pace on the measured course. This allowed us to map the individual movement patterns of the participants and subtle movement variations. The MVN Analyze software then processes, analyses, and displays this data in real time, enabling accurate movement analysis [36,37,38]. The selected gait parameters provide a comprehensive description of spatiotemporal gait patterns and postural control, which are sensitive indicators of motor recovery after stroke. IMU-based systems like MVN Awinda have demonstrated high validity and reliability for clinical gait assessment and allow detection of subtle asymmetries or compensatory strategies that might not be evident during visual gait observation.

### 2.5. Interventions

Each group attended five sessions a week for 3 weeks. Each session was 30 min long, including a 5-min warmup, a 20-min main session according to the therapy of the group, and a 5-min cooldown. In all three groups, the rate of perceived exertion (RPE) was used to maintain high training intensity, which was additionally monitored by heart rate using Polar RS800CX HR watches (Polar Electro Oy, Kempele, Finland). After each training session, participants were asked to rate their perceived exertion on the 20-point Borg scale [39]. The combined use of RPE and heart rate is considered a valid and practical method for regulating exercise intensity in stroke rehabilitation. All participants completed the training sessions as planned; no dropouts occurred and no unexpected adverse events were reported during the 3-week period, indicating that all three interventions are safe and feasible therapies for stroke patients. The intervention period was limited to three weeks, aiming to assess short-term functional effects rather than long-term neuroplasticity.

#### 2.5.1. Robotic Therapy

**Figure 2:**
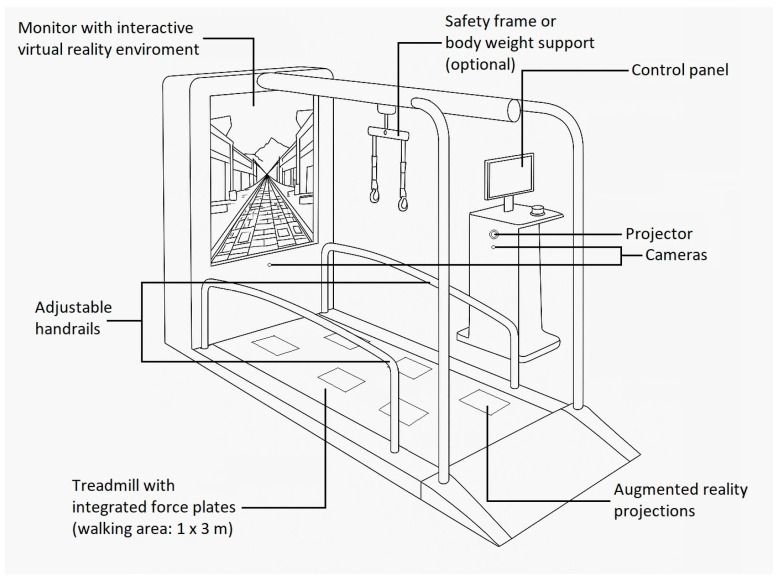
C-Mill VR+ setup for robotic therapy.

We used C-Mill VR+ (Motek Medical B.V., Amsterdam, The Netherlands) to implement robotic therapy (Figure 2). The C-Mill VR+ system is a 3 m long and 1 m wide treadmill with a force platform underneath. In front of the treadmill, a 65” fixed-position monitor provides the virtual reality environment, and a projector located next to the treadmill allows various tasks to be projected onto the treadmill’s ribbon, with immediate real-time feedback to the patient and therapist via the force platform. The system is controlled from a PC running Windows 10-64-bit using CueFors (2.6.4) software. The computer provides the therapist with real-time detailed data on the patient’s COP path and gait parameters, which are reported in detail after the task is completed, allowing for personalized therapy. The tool allows the implementation of several types of exercises: balance exercises, goal-directed stepping with or without obstacles, obstacle crossings, gait acceleration and deceleration by maintaining position within a projected walking area that moves along the treadmill, tandem stepping, step length and step phase manipulation, gait pace manipulation with auditory stimuli, and adaptive gait modulation task. Based on the feedback from the system, the therapist can tailor the training to the individual by increasing/decreasing the speed of the treadmill, the number/size of obstacles, the available reaction time, and the difficulty of the task.

In the first half of the first session, patients completed traditional treadmill training on the device to get used to walking safely on the treadmill. We then introduced a goal-directed stepping exercise, where the patient has to step on predetermined places, thus promoting gait adaptation. From the second session onwards, we gradually manipulated step length, step width, symmetry, and speed during the task to suit the patient’s individual condition. The visually guided steps could be made more difficult by increasing the irregularity of the target sequence, or by adjusting the size of the targets to the participant’s shoe size or adding obstacles. Once the task was smooth for the patient, we started to introduce the additional therapeutic programs provided by the device as mentioned above, such as obstacle crossing, tandem walking, slalom walking, using auditory stimuli to manipulate the rhythm of the gait, manipulating the length of the step phases, etc. Before the cooldown, the sessions were all concluded by an adaptive gait modulation task, during which the patient had to avoid different objects while touching specific points on the treadmill while walking. The difficulty of each therapy session was always adjusted to the patient’s current condition, the choice of tasks was based on the patient’s individual abilities and disabilities, and the difficulty of the tasks was set accordingly and fine-tuned based on feedback from the C-Mill system during the tasks by a trained physiotherapist. The intensity of the training was easily adjusted by setting the treadmill speed, adjusting the difficulty of the tasks, and selecting the appropriate types of programs [40,41,42].

#### 2.5.2. Agility Training

During the agility training, the participants performed gait training, coordination training, balance training, posture correction exercises, and muscle strengthening with and without the use of different tools (Dynair cushion, Bosu, fit-ball, Pilates ball, weight ball, end-weighted stick, TRX, coordination ladder, and barriers). This block also included different surface modifications and direction changes during the tasks and manipulation of the speed of the tasks, as well as the use of height stimuli. The difficulty of the tasks was adjusted each time according to the participants’ improved performance. The movements used here required high-order attention and executive functions as well as high-speed cognitive processing speed in response to visual and auditory cues. The training provided a strong neuromuscular stimulus due to the constantly changing sensory environment, such as soft/hard surface tasks, heavy/light weighted tools, slow/fast movement executions, and reflexive response/conscious response to external stimuli during task.

The agility training (AG) protocol was organized into three progressive phases to ensure consistency while allowing therapist adaptation based on individual performance. Each session lasted 30 min (including warmup and cooldown), and the structure was designed to match the training intensity of the robotic (ROB) and exergaming (EX) interventions.

- Sessions 1–2: Emphasis was placed on establishing fundamental static and dynamic balance and ensuring safety of movement. Participants practiced supported standing, weight transfers, sit-to-stand transitions, and basic gait initiation tasks to build stability and confidence.
- Sessions 3–6: Focus shifted to the development of coordination, reaction time, and movement accuracy. Exercises included slalom walking, step variations in multiple directions, simple obstacle courses, and the use of tools such as Dynair cushions, Bosu, fit-balls, coordination ladders, and light weights. Directional changes and variable speeds were gradually introduced to promote adaptability.
- Sessions 7–15: Advanced agility and dual-task training were introduced. Participants performed complex postural control and reaction exercises, including direction changes on auditory or visual cues, multi-step sequences on unstable surfaces, and combined upper- and lower-limb strengthening tasks. Cognitive elements were integrated into motor performance (e.g., responding to verbal prompts and memorizing movement patterns) to enhance executive control during movement.

Task difficulty, movement speed, and balance demands were progressively increased while maintaining comparable session duration and perceived exertion (RPE) levels across participants. The progression was guided by a physiotherapist to ensure safety and task adaptation to each participant’s functional capacity [4,6,7].

#### 2.5.3. Exergaming

**Figure 3:**
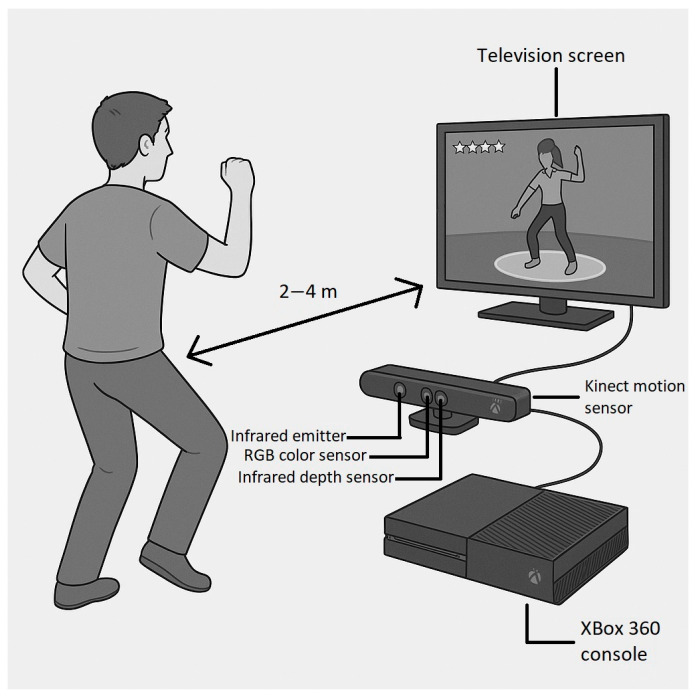
Kinect-based motion tracking setup for exergaming.

Gamification (also known as serious gaming) is a method to use gaming scenarios and methods for scientific purposes. In the gamified environment, challenging tasks help to maintain motivation and increase involvement in the process, especially if repetitive and long procedures are used. In the case of physical rehabilitation, extensive body movements are involved in so-called exergames. Serious gaming in general can contribute to the rehabilitation of stroke patients. For the experiment, the Microsoft Xbox 360 system and Kinect motion sensor (Microsoft Corporation, Redmond, WA, USA) were used (Figure 3). Despite the fact that newer versions and updates are available, this setup fulfilled the requirements. Movements, body posture, and even face gestures can be detected using RGB and infrared cameras. In addition, depth mapping is also available. Three different VR programs were used during the intervention: Kinect Adventure—Reflex Ridge, Kinect Adventure—Space Pop, and Just Dance.

During Reflex Ridge, the participant’s virtual avatar stands on a platform moving on a track, which, after starting, continuously moves forward while obstacles appear from different directions. The visual stimuli provoke reflexive and voluntary avoidance movements, such as weight shifting, stepping sideways, squatting, and jumping up. The avatar executes the avoidance movements performed by the participant in real time in real space, thus avoiding collisions in virtual space. The system provides scores based on the obstacles avoided, so it is easy to monitor the patient’s performance and adjust the appropriate load. The difficulty level can be increased by increasing the number and density of obstacles in the virtual space until the optimal load is reached.

Space Pop can be understood as a kind of complex target exercise, which requires the flawless execution of complex movements to be successful. The participant moves his avatar back and forth and side to side in these directions, and up and down with specific arm movements, while touching different targets in all dimensions of virtual space. The more targets one touches, the more points one scores at the end of the task. During the task, the participant performs complex spatial movements based on quick reactions and changes in direction through targeted movements.

In Just Dance, the participant’s task is to mimic as closely as possible the ever-changing complex rhythmic movements on the screen. The task requires a fast motor response to incoming visual and acoustic stimuli and precise execution. The program assigns scores between 0 and 10,000 based on the accuracy of the movement [6,43,44,45,46].

### 2.6. Statistical Analysis

Data were expressed as mean ± standard deviation (SD). The normal distribution of variables was assessed using the Shapiro–Wilk test. Incomplete data accounted for less than 2% of all measurements and were handled by pairwise deletion to retain the maximum number of valid observations for each statistical test. Multiple imputation was not applied because the amount of incomplete data was minimal and the sample size limited. Extreme values were screened using boxplots and standardized z-scores (>3 SD from the group mean). Potential outliers were cross-checked with the original measurement sheets, and biologically implausible values were excluded only if verified as recording or entry errors. The choice of statistical tests followed standard methodological criteria: parametric tests (*t*-test, ANOVA, and ANCOVA) were used for normally distributed data with homogeneity of variance, whereas non-parametric tests (Wilcoxon signed-rank and Kruskal–Wallis) were applied when normality or homoscedasticity assumptions were not met. Effect size reporting occurred. Within-group changes were summarized using the standardized response mean (SRM = mean change/SD of change) and Hedges’ g_av with small-sample correction. For between-group comparisons of change, effect sizes were derived from the ANCOVA models: partial η^2^ for omnibus group effects and Hedges’ g for adjusted pairwise differences in change (difference-in-differences using estimated marginal means). All effect sizes are reported with 95% confidence intervals [47].

Based on normality, one-way ANOVA or the non-parametric Kruskal–Wallis test was applied to compare the ROB, AG, and EX groups at baseline and post-intervention. The extent of within-group changes (pre- vs. post-test) was assessed using paired *t*-tests for normally distributed variables and Wilcoxon signed-rank tests for non-normal distributions. Between-group comparisons of change scores were tested using one-way ANOVA or Kruskal–Wallis, with Tukey’s HSD post hoc test used for multiple comparisons (*p* < 0.05). To examine the magnitude of within-group changes over time, Cohen’s d was calculated (very small = 0.01; small = 0.20; medium = 0.50; large = 0.80; very large = 1.20; huge = 2.00). Group-level comparisons were carried out for gait parameters (e.g., speed, cadence, step length/width, stance, and swing phases), posturography variables (COP Sway, area, and velocity in WEO, WEC, NEO, and NEC), balance scales (BBS and BESTest), functional measures (mRS, BI, SE-ADL, and FIM), walking performance (6MWT and 10mWT), and rate of perceived exertion (RPE). All statistical analyses were conducted using Microsoft Excel 2010 and R software (version 4.3.0). To control for baseline differences in primary gait parameters (e.g., walking speed), ANCOVA was used with pre-treatment values as covariates. Because baseline differences were observed in the 10-m walk test (10mWT, *p* = 0.0499) and the center of pressure (COP) WEC Sway parameter (*p* < 0.0001), these variables were included as covariates in the analysis of corresponding post-intervention outcomes. Accordingly, an ANCOVA model was applied for post-training comparisons of gait and balance outcomes, with baseline scores entered as covariates. Both unadjusted (ANOVA) and adjusted (ANCOVA) results are presented to enable transparent interpretation. No correction for multiple comparisons was applied due to the exploratory nature of the study, and findings should be interpreted accordingly. Nevertheless, the potential inflation of Type I error due to multiple testing was acknowledged, and the interpretation of *p*-values was conducted with caution. For reference, if a conservative Bonferroni correction (α = 0.05/40 ≈ 0.00125) were applied, only the most robust between-group effects (e.g., COP WEC Sway and 10mWT) would remain significant, confirming that the main conclusions are not dependent on marginal significance thresholds. Given the limited sample size (n = 33; 11 participants per group), a post hoc power estimation was conducted using G*Power (version 3.1). Assuming an α = 0.05 and an observed effect size of η^2^ = 0.25 (Cohen’s f = 0.577) for key gait and balance parameters (e.g., 6MWT and BBS), the achieved power (1−β) was approximately 0.42–0.58. This indicates that the study was underpowered to detect small-to-moderate between-group effects, and results should therefore be interpreted with caution. The study was not designed as a pilot or feasibility trial but represents a complete comparative investigation based on the available patient cohort. The relatively small sample size (n = 33; 11 per group) inevitably limited the statistical power for between-group comparisons. The post hoc power analysis (1−β = 0.42–0.58) confirmed that the study was underpowered to detect small-to-moderate effects. Therefore, the statistical findings were interpreted cautiously, and no claims of superiority were made. The analyses should be regarded as exploratory and intended to inform the design of future adequately powered trials.

## 3. Results

### 3.1. Baseline Characteristics

A total of 33 people were selected for our study based on the criteria and randomly allocated in a 1:1:1 ratio to the ROB (female, *n* = 4; male, *n* = 7), AG (female, *n* = 4; male, *n* = 7), and EX (female, *n* = 4; male, *n* = 7) groups. The mean time since the stroke was 5 weeks in each group. The physical characteristics (age, height, and weight) and the initial results of the participants are summarized in Table 2, sorted by group. Examining the data, no significant differences were found between the three groups in terms of time since stroke, MMSE score, age, and height (all *p* > 0.05). RPE values were collected during the 15 days of the interventions (Figure 3).

**Figure 4:**
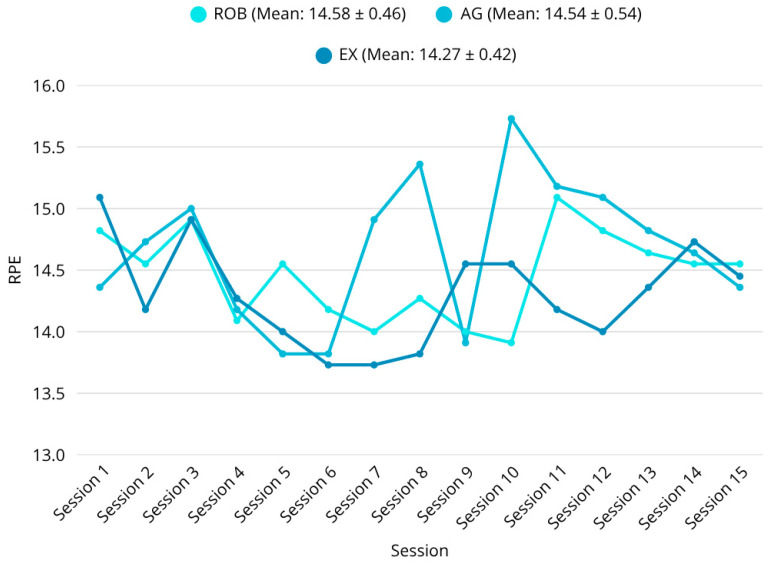
RPE values during the interventions. No significant difference in mean RPE (*p* > 0.05).

The mean RPE value was 14.58 (±0.46) in the ROB group, 14.54 (±0.54) in the AG group, and 14.27 (±0.42) in the EX group (Figure 4). There was no statistically significant difference in RPE between the three groups (*p* > 0.05). Significant differences were detected between the groups in the initial scores of the 10mWT (F = 3.32; *p* = 0.0499) and COP WEC Sway (F = 95.44; *p* < 0.0001). No statistically significant differences between groups were detected for the other initial outcomes.

### 3.2. Functional Abilities

**Figure 5:**
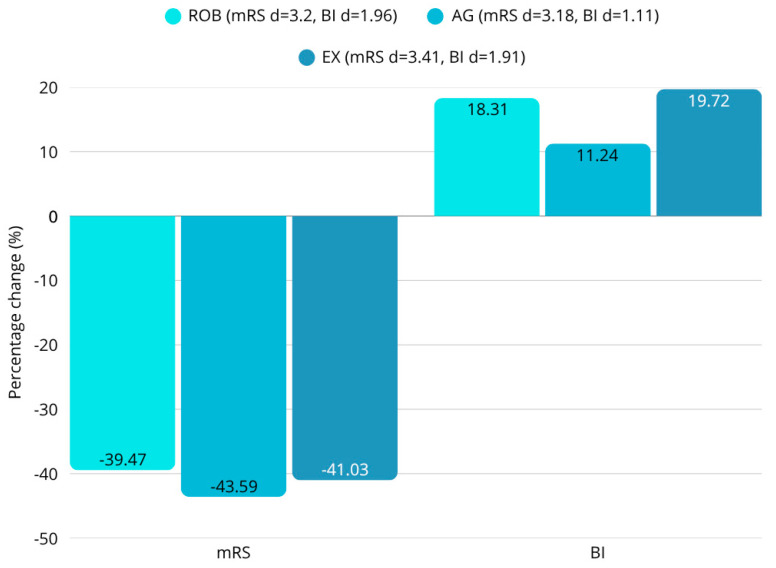
Percentage change in scores on mRS and BI. Significant improvements in all groups (all *p* < 0.001). No significant differences between groups (all *p* > 0.05).

Scores on the mRS (ROB: d = 3.2; AG: d = 3.18; EX: d = 3.41), BI (ROB: d = 1.96; AG: d = 1.11; EX: d = 1.91), SE-ADL (ROB: d = 0.70; AG: d = 0.84; EX: d = 0.72), FIM (ROB: d = 3.12; AG: d = 2.53; EX: d = 2.77), and FIM motor subscale (ROB: d = 2.84; AG: d = 2.64; EX: d = 3.30) showed significant improvements in all three groups (all *p* < 0.001) (Figure 5, Table 3). No significant differences were found between the groups in mRS, BI, SE-ADL, FIM, and FIM motor subscale (all *p* > 0.05).

### 3.3. Balance and Postural Stability

**Figure 6:**
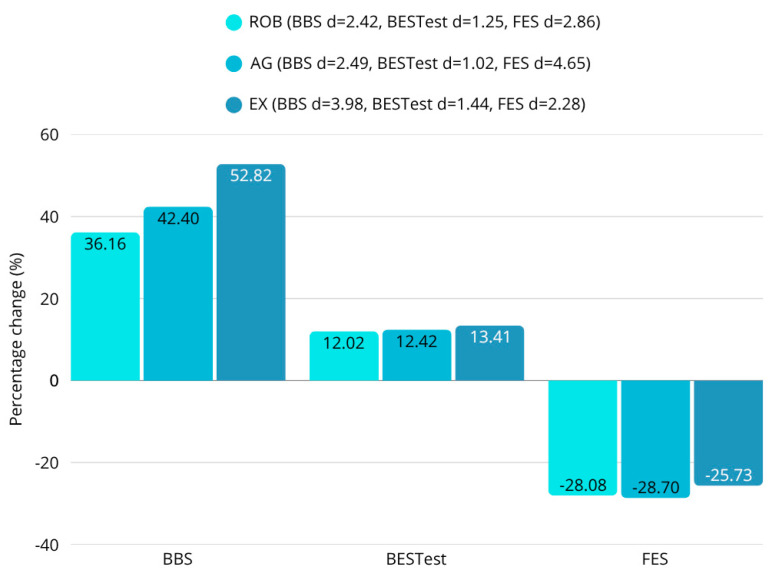
Percentage change in scores on balance-related scales. Significant improvements in all groups (all *p* < 0.001). Highly significant improvement in BBS between EX and ROB (diff = 0.364; *p* = 0.0001), EX vs. AG (diff = 6.364; *p* = 0.0003), and AG vs. ROB: n. s. (diff = 0.364; *p* = 0.964).

BBS (ROB: d = 2.42; AG: d = 2.49; EX: d = 3.98), BESTest (ROB: d = 1.25; AG: d = 1.02; EX: d = 1.44), and FES (ROB: d = 2.86; AG: d = 4.65; EX: d = 2.28) scores improved significantly in all groups (all *p* < 0.001) (Figure 6, Table 3). Comparing the groups, we found that there was a statistically significant difference in the scores for BBS after the intervention (F = 14.32; *p* < 0.001).

A highly significant improvement in BBS was observed between the EX and the ROB (diff = 6.727; *p* = 0.0001 **), indicating that the exergaming group showed substantial balance gains compared to the robotic rehabilitation group. The difference between the EX and the AG was statistically significant (diff = 6.364; *p* = 0.0003); the exergaming group showed a trend toward better balance performance. The difference between the AG and the ROB was not statistically significant (diff = 0.364; *p* = 0.964). The COP Sway results for the four body positions tested showed significant improvement for the ROB group for WEO, WEC, and NEC (all *p* < 0.001) (Table 3). The magnitude of the effects is summarized in Table 4.

The EX group showed highly significant improvements for WEO and NEC (*p* < 0.001) and significant improvement for NEO (*p* < 0.05). The AG group showed significant improvement only for WEO (*p* < 0.001). Comparing the groups, we found that there was a statistically significant difference for WEC after the intervention (F = 10.64; *p* < 0.001). A statistically significant improvement in postural stability was observed between the ROB and the AG (diff = −4.53 mm; *p* = 0.0003), reinforcing the effectiveness of agility-based interventions. A significant difference was also detected between the EX and the ROB (diff = −3.31 mm; *p* = 0.0077), supporting the potential benefits of exergaming therapy in balance recovery. The COP Velocity results showed a highly significant change in all four test positions for the ROB and AG groups (all *p* < 0.001). For the EX group, a highly significant change was observed for NEO and NEC (all *p* < 0.001), while a significant change was observed for WEO (*p* < 0.05). Comparing the groups, we found no statistically significant difference in the results of COP Velocities. The COP Area result showed a highly significant change in the ROB and AG groups for WEO and NEC (all *p* < 0.001) and a significant change for NEO (*p* < 0.05). For the EX group, a highly significant change was observed for NEC (all *p* < 0.001). No significant differences were found between groups when comparing the results of COP Area.

### 3.4. Gait Assessments

**Figure 7:**
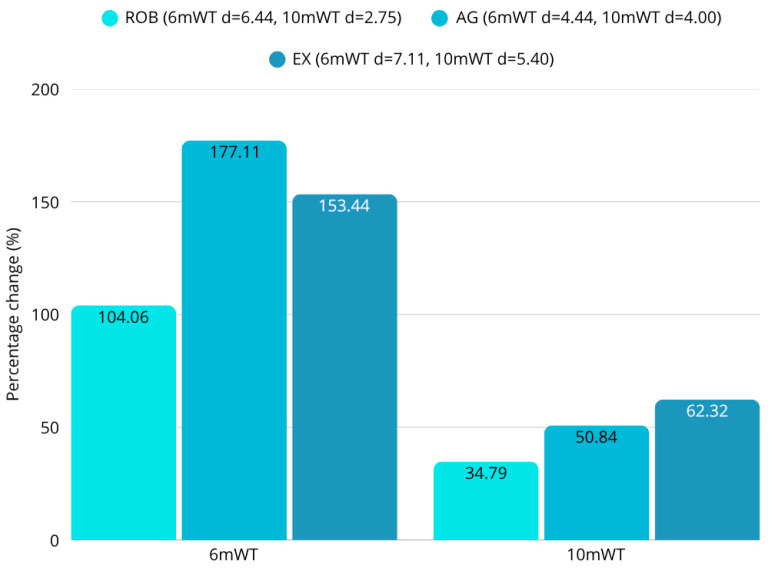
Percentage change in 6mWT and 10mWT. Significant improvements in all groups (all *p* < 0.001). Significant difference for 6mWT (F = 6.80; *p* < 0.01). Significant ROB vs. AG (diff = 62.27; *p* = 0.0044) and EX vs. AG (diff = 50.45; *p* = 0.02272).

The 6mWT (ROB: d = 6.44; AG: d = 4.44; EX: d = 7.11) and 10mWT (ROB: d = 2.75; AG: d = 4.00; EX: d = 5.40) showed significant improvements in all three groups (all *p* < 0.001) (Figure 7, Table 3). Comparing the groups, significant differences were found between the groups for the 6mWT results (F = 6.80; *p* < 0.01). A statistically significant improvement in 6mWT was observed between ROB and AG groups (diff = 62.27; *p* = 0.0044) and EX and AG groups (diff = 50.45; *p* = 0.02272), supporting the benefits of agility training endurance recovery.

**Figure 8:**
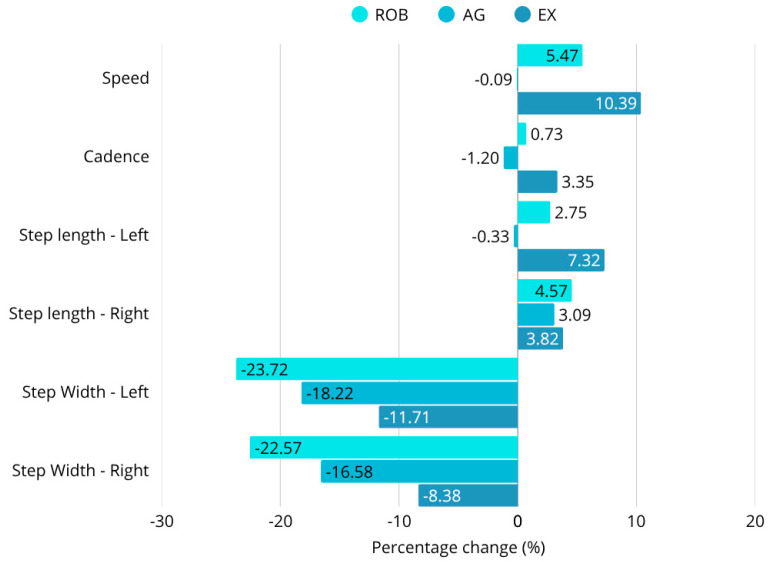
Percentage change in gait parameters. ROB: significant improvements in number of steps (*p* < 0.001) and step width (*p* < 0.05). EX: significant improvement in left step length, swing phase, stance phase, double support phase, and total gait cycle (*p* < 0.05). AG: significant improvement in double support phase—right (*p* < 0.05). Significant group differences: swing phase left (F(2,30) = 3.61; *p* = 0.0393 and swing phase diff (F(2,30) = 3.35; *p* = 0.0485). Significant difference between AG and EX in swing phase diff (*p* < 0.05).

Based on 3D motion analysis, the ROB group showed significant improvements in both number of steps (*p* < 0.001) and step width (*p* < 0.05) with both right and left feet. The EX group showed a significant improvement in left step length as a result of the therapy (Figure 8). Between-group comparisons were adjusted using ANCOVA to account for baseline differences.

In terms of gait cycle phases, the EX group showed improvements in left swing phase, left stance phase, and double support phase left and right scores, as well as in total gait cycle data (all *p* < 0.05). The AG group only showed significant improvement in double support phase right (*p* < 0.05) (Table 5). Comparing the results between the groups, statistically significant differences were observed between the groups for the swing phase left (F(2,30) = 3.61; *p* = 0.0393) and swing phase difference (F(2,30) = 3.35; *p* = 0.0485). A significant difference was detected between the AG and the EX groups in terms of swing phase difference (*p* < 0.05).

## 4. Discussion

This study aimed to compare the clinical effectiveness of three distinct high-intensity rehabilitation modalities—robotic-assisted therapy, screen-based interactive exergaming, and conventional agility training—on gait function, postural control, and overall mobility in individuals recovering from subacute ischemic stroke. The results provide strong evidence that technology-assisted rehabilitation, particularly robotic and exergaming interventions, can significantly improve spatiotemporal gait parameters, balance performance, and functional outcomes over a relatively short intervention period. Although the within-group improvements reached statistical significance in several outcome measures, the between-group differences were generally weak or non-significant. For instance, in key functional indicators such as the mRS, BI, and FIM, no statistically significant group differences were observed. Given the relatively small sample size (n = 11 per group), these findings should be interpreted with caution. The observed between-group differences—some of which fall close to the conventional significance threshold (e.g., *p* = 0.041 and *p* = 0.049)—may reflect statistical fluctuations rather than robust intervention effects. Therefore, claims of superiority among the studied interventions are not fully supported by the data and should be considered exploratory. Given the limited power and small sample size, the results should be viewed as indicative rather than confirmatory and serve primarily to guide future large-scale investigations. Future research involving larger adequately powered samples is warranted to validate these trends and better understand differential intervention effects [48,49].

The robotic therapy group showed the most pronounced gains in multiple key gait parameters, including walking speed, step length, and stance phase symmetry. Walking speed increased by 5.4%, and step width decreased by 23.7%, indicating enhanced locomotor efficiency and reduced lateral instability. The improvements in stance duration and step symmetry also reflected a shift toward more coordinated, efficient gait patterns. These results support previous findings that robotic therapy can facilitate neuroplastic adaptation and motor relearning through intensive task-specific repetition [6,7,40,50,51].

The exergaming group demonstrated significant gains in cadence, postural sway reduction, and balance scores. For example, highly significant sway amplitude reductions were observed in eyes-closed postural conditions (e.g., WEC: F = 95.44; *p* < 0.0001), and cadence improved markedly. These changes may be attributed to the interactive and multisensory nature of virtual reality tasks, which are known to promote attentional focus and sensorimotor coordination [43,44,45,46]. The exergaming group also exhibited a 10.4% increase in walking speed, improved Berg Balance Scale (BBS) scores, and favorable changes in the Functional Independence Measure (FIM), suggesting a broad functional benefit with strong motivational effects.

In contrast, agility training resulted in more modest improvements. Step width decreased by 18.2%, but there were no significant changes in walking speed, cadence, or step symmetry. These results imply that conventional agility-based therapy may be more suitable for functional maintenance rather than facilitating neuroplastic recovery. This aligns with prior studies suggesting that agility programs are effective for preserving existing mobility but are limited in their capacity to stimulate substantial neurological change [52,53].

Statistical analyses using both parametric and non-parametric methods confirmed the robustness of these findings. ANOVA revealed significant between-group differences for key gait parameters such as walking speed (F = 3.44; *p* = 0.0453), step duration (F = 5.62; *p* = 0.0085), and gait symmetry. Post hoc Tukey-HSD tests further demonstrated that most significant differences occurred between the exergaming and agility groups. Within-group comparisons also showed statistically significant improvements in the robotic and exergaming groups across multiple domains, including walking performance (6MWT: F = 6.80, *p* = 0.0037; 10mWT: F = 3.32, *p* = 0.0499) [54,55]. Very large within-group improvements were observed in walking endurance across all the groups (SRM and Hedges’ g_av in the large range), but comparative effects were modest; the adjusted between-group effect sizes from ANCOVA (partial η^2^ and pairwise Hedges’ g) are reported in Table 3 and did not indicate unequivocal superiority. Replication in larger and more diverse cohorts is required to confirm these findings. Although both robotic and exergaming interventions achieved 5–10% improvements in gait speed, these changes may not fully exceed the minimal clinically important difference (MCID) for post-stroke gait rehabilitation. Recent evidence suggests that an MCID of approximately 0.08–0.10 m/s represents a meaningful improvement in chronic stroke patients. Therefore, while the observed improvements are encouraging and consistent with functional gains, their clinical relevance should be interpreted with caution until confirmed in larger adequately powered trials [56]. Notably, perceived exertion, measured via the RPE scale, was significantly lower post-intervention in the exergaming group during several training timepoints (e.g., RPE (session 10): ROB vs. AG, *p* = 0.0067; EX vs. AG, *p* = 0.0372). This suggests a more favorable engagement and fatigue profile, which may support better adherence and long-term implementation [57,58].

The strength of this study lies in its multidimensional outcome assessment strategy. Objective 3D gait analysis (Xsens MVN), posturography, clinical scales (e.g., BBS, BESTest, FIM, mRS, and BI), and patient-reported exertion levels were all included, providing a comprehensive evaluation of therapy effectiveness. The use of both spatiotemporal and subjective measures enables deeper insight into the mechanisms of functional change.

Despite some deviations from normality—particularly in step asymmetry and postural sway values—these variations reflect real-world heterogeneity in post-stroke recovery. Such findings suggest that robotic and VR-based interventions may be adaptable to a broad spectrum of patients with differing gait profiles. The presence of statistically significant and clinically relevant improvements across multiple outcomes, even with this variability, supports the real-life applicability of these interventions.

From a clinical perspective, robotic therapy appears to be best suited for patients with moderate to severe gait impairments who need high-repetition intensity-controlled interventions to restore symmetry and speed. Exergaming provides a practical and motivating alternative, particularly in environments where access to robotic systems is limited. The favorable RPE and balance outcomes further highlight its potential for outpatient and home-based use [51]. Agility training, while less effective in enhancing key gait metrics, may still play a valuable role in stabilizing functional capacity in later recovery phases or for individuals who have already regained a degree of autonomy [52,59,60].

These results are aligned with current trends in neurorehabilitation that advocate for individualized technology-supported intervention plans adapted to specific functional profiles. Future applications may include adaptive algorithms, artificial intelligence, and remote monitoring, further enhancing the scalability and precision of neurorehabilitation programs [61].

Beyond statistical interpretation, the clinical applicability of these findings warrants consideration. The three high-intensity interventions studied here offer distinct benefits in real-world rehabilitation. Robotic-assisted therapy may be particularly suitable for severely impaired patients who require intensive guided motion with minimal compensatory movement. VR-based exergaming, by contrast, may be more engaging and feasible in outpatient or home-based programs due to its portability and motivational design. Beyond clinical efficacy, the economic and practical feasibility of implementing advanced rehabilitation technologies plays a decisive role in their widespread adoption. Robotic-assisted systems such as the C-Mill VR+ involve substantial initial investment and maintenance costs, which may limit accessibility in smaller hospitals or low-resource settings. However, these technologies can also reduce therapist workload, provide objective performance tracking, and potentially shorten recovery timelines, offering long-term economic benefits when integrated into multidisciplinary rehabilitation programs [58]. In contrast, exergaming platforms—based on commercially available hardware such as Microsoft Kinect—are significantly more affordable and portable, making them attractive for home-based or outpatient rehabilitation [62]. Their lower cost and ease of deployment could enable scalable solutions, especially in regions where access to robotic therapy is limited. Nevertheless, both approaches require proper patient supervision, therapist training, and infrastructure support to ensure safety. Agility training, although conventional, remains broadly accessible and easy to tailor to patient needs. These characteristics underscore the importance of individualizing therapy choices not only based on clinical scores but also on contextual, economic, and psychosocial factors.

The superior performance of robotic and exergaming interventions compared to conventional agility training can be interpreted within the framework of experience-dependent neural plasticity. Robotic-assisted rehabilitation delivers high-intensity, repetitive, and precisely controlled movement patterns, which are key stimuli for inducing synaptic plasticity and cortical remapping in motor-related brain regions. The continuous real-time feedback provided by the robotic system augments proprioceptive input and reinforces sensorimotor integration, leading to improved gait symmetry and coordination. Similarly, exergaming-based interventions engage multimodal sensory feedback (visual, auditory, and proprioceptive) and cognitive–motor coupling, which may stimulate broader neural networks involved in balance and attention regulation. This multisensory engagement enhances motivation and adherence, which are also known to positively influence neuroplastic recovery processes. In contrast, agility training, while beneficial for functional maintenance and endurance, may lack the precise repetition and quantitative feedback required to drive robust neural reorganization. Its benefits likely stem from improved musculoskeletal strength, balance adaptation, and compensatory motor strategies rather than from cortical-level plastic changes. These mechanisms together explain why robotic and exergaming interventions achieved more pronounced improvements in gait and gait-related balance metrics in the present study [19,63]. Further studies in larger cohorts and diverse clinical environments are needed to validate these insights and support implementation strategies. Due to the small sample size, this study should be considered exploratory. Larger randomized controlled trials are needed to validate these findings and explore long-term clinical effects. However, these observations should be interpreted with caution given the limited sample size and moderate statistical power, which restrict the generalizability of the between-group findings.

This study had several limitations. The sample size (*n* = 33) was relatively small, which may have limited the detection of subgroup-specific effects. The intervention period was short (three weeks), and no long-term follow-up was conducted, preventing conclusions regarding the durability of the observed improvements. Although the inclusion criteria were designed to ensure safety and homogeneity, they may reduce generalizability to more complex stroke populations, such as those with recurrent events, severe cognitive deficits, or multiple comorbidities. The variability in baseline characteristics—especially in the agility and exergaming groups—may have influenced certain outcome distributions, which necessitated the use of non-parametric analysis for some variables. This variability is also indicative of real-world clinical diversity and contributes to the ecological validity of the findings. It should be noted that, unlike the robotic and exergaming interventions, the agility training protocol relied on therapist-guided progression and individualized task adjustment rather than a fixed software-controlled structure. Although this design reflects real-world clinical practice and allows for patient-specific customization, it also introduces variability in task intensity and content. This reduced standardization may represent a potential confounding factor that could affect the direct comparability of outcomes between interventions. Future studies with larger samples should consider implementing a more structured agility training framework or digital tracking to enhance protocol consistency.

## 5. Conclusions

Within the limitations of this pilot-scale study, these technology-driven rehabilitation methods (robotic therapy and exergaming) appear to facilitate greater short-term improvements in post-stroke gait parameters compared to traditional agility training. The combination of statistical analyses (ANOVA, post hoc Tukey-HSD, *t*-tests, Wilcoxon, and normality tests) consistently supported the hypothesis that robotic therapy and exergaming facilitate greater improvements in walking speed, step length, and balance-related gait metrics. No significant differences were found between groups in the results of most scales, but the objective instrumental tests revealed several differences. While robotic therapy achieved a 5% improvement in gait speed and exergaming a 10% improvement, the speed of the agility training of participants was essentially unchanged (0.1% decrease). In terms of changes in step length, the improvement in the robotic group was 3–5%, in the exergaming group 4–7%, and in the agility group 0–3%. In terms of step width, the 23–24% reduction in robotic therapy also exceeded the 17–18% reduction in agility training. A similar hierarchical arrangement of results was observed for postural instability. Across the four body positions measured, the COP pathway decreased by an average of 40% for robotic therapy, 32% for exergaming, and 28% for agility therapy. Furthermore, the 53% improvement in static and dynamic balance in the exergaming group significantly outweighed the 42% and 36% improvements in the agility and robotics groups. However, patients’ endurance showed a greater improvement with agility therapy (177%) than with either robotic (104%) or exergaming (153%). These findings have profound implications for stroke rehabilitation, advocating for the prioritization of robotic and exergaming interventions over conventional functional maintenance therapies like agility training.

Future research should explore longitudinal effects and patient-specific predictors of rehabilitation success, further refining individualized rehabilitation strategies for optimizing stroke recovery. In conclusion, this study provides evidence that robotic and exergaming therapies can deliver substantial improvements in gait and balance for stroke patients. Future research should validate these results in larger samples and with extended follow-up periods.

### Limitations

While this study confirms the short-term effectiveness of robotic and exergaming interventions in improving gait and balance, it remains unclear whether these benefits are maintained in the long term. Future studies should incorporate longitudinal follow-ups to assess the retention of motor function and to identify which rehabilitation modality yields the most lasting improvements. The study involved 33 participants. While this may be sufficient to find statistically significant results, a larger sample size would increase the statistical power of the study and further enhance the generalizability of the findings. We were unable to assess the long-term additional costs of individual therapeutic interventions, which could also form the basis of future research. A detailed cost-effectiveness analysis (e.g., cost per FIM gain, therapist time, and equipment amortization) is recommended for future larger-scale studies. We used strict exclusion criteria to reduce variability within groups and ensure participant safety during high-intensity exercise programs. However, these strict criteria limit the generalizability of our findings to the broader stroke population, which often presents with multiple comorbidities. Further research is needed to determine how applicable the results are to a wider range of stroke patients. The lack of correction for multiple testing increases the risk of false positives and limits the strength of the conclusions. Future confirmatory studies should apply appropriate statistical corrections. The limited sample size (*n* = 33; 11 per group) reduced the statistical power of between-group comparisons. Although not designed as a pilot study, the available patient cohort constrained recruitment. A post hoc power estimation (1−β ≈ 0.42–0.58) indicates that some true effects may not have reached statistical significance; therefore, the findings should be interpreted with caution. The initially high within-group effect sizes (e.g., Cohen’s d) were re-evaluated and recalculated using standardized response means and Hedges’ g with small-sample correction to avoid inflation due to variability or outliers. Comparative between-group effects were expressed through ANCOVA-adjusted effect sizes (partial η^2^; Hedges’ g), which confirmed consistent trends without evidence of overestimation. The exploratory nature of the study and the large number of outcome variables increase the risk of Type I error; therefore, the findings should be interpreted cautiously, although the most robust effects (e.g., COP WEC Sway and 10mWT) remained consistent under conservative thresholds.

## Figures and Tables

**Table 1:** Participants’ characteristics.

		ROB		AG		EX	
	Variable	Mean, n	SD, %	Mean, n	SD, %	Mean, n	SD, %
Physical characteristics	Age (y, SD)	66.4	4.78	67.0	3.22	65.7	4.38
	Height (cm, SD)	173.1	6.32	174.7	7.77	171.4	7.59
	Mass (kg, SD)	71.9	7.05	76.0	11.20	72.2	5.00
	BMI (kg/m^2^, SD)	24.0	2.44	24.9	2.96	24.7	3.01
Sex	Male (n, %)	4	36	3	27	4	36
	Female (n, %)	7	64	8	73	7	64
Affected brain area	Right hemisphere (n, %)	2	18	3	27	3	27
	Left hemisphere (n, %)	8	73	7	64	4	36
	Cerebellar (n, %)	1	9	1	9	4	36
	Brainstem (n, %)	0	0	0	0	0	0
Comorbidity	Hypertension (n, %)	1	9	1	9	5	45
	Ischemic heart disease (n, %)	3	27	0	0	3	27
	Atherosclerosis (n, %)	1	9	3	27	0	0
	Diabetes (n, %)	3	27	3	27	0	0
Destructive habits	Smoking (n, %)	8	73	9	82	7	64
	Alcohol (n, %)	3	27	3	27	0	0

**Table 2:** Group comparison at baseline.

	ROB		AG		EX		All	
Variable	Mean	SD	Mean	SD	Mean	SD	Mean	SD
Age (y)	66.4	4.78	67.0	3.22	65.7	4.38	66.4	4.13
Height (cm)	173.1	6.32	174.7	7.77	171.4	7.59	173.1	7.23
Mass (kg)	71.9	7.05	76.0	11.20	72.2	5.00	73.4	7.75
mRS (point)	3.5	2.09	3.6	0.52	3.6	0.52	3.5	1.04
BI (point)	76.4	5.05	64.7	5.46	64.6	8.20	68.6	6.24
FIM (point)	66.5	6.83	70.3	5.62	69.3	7.16	68.7	6.54
FIM-MS (point)	51.6	7.22	51.7	8.11	51.0	7.27	51.4	7.53
SE-ADL (%)	71.8	13.28	67.3	7.86	70.9	12.21	70.0	11.12
BBS (point)	20.4	2.58	19.7	3.58	22.6	2.54	20.9	2.90
FES (point)	81.3	5.92	82.4	5.24	80.9	6.17	81.5	5.78
BESTest (point)	72.6	8.79	71.0	11.24	71.2	7.81	71.6	9.28
6mWT (m)	123.2	16.77	113.2	14.37	119.1	19.06	118.5	16.73
10mWT (m/s)	0.9	0.14	0.8	0.12	0.8	0.08	0.9	0.11
COP WEO Sway (mm)	12.2	3.37	15.4	6.03	13.7	3.91	13.8	4.44
COP WEC Sway (mm)	13.2	2.95	3.2	0.97	4.0	1.00	6.8	1.64
COP NEO Sway (mm)	12.2	5.47	8.2	4.09	8.3	3.61	9.5	4.39
COP NEC Sway (mm)	13.7	2.88	10.1	6.40	12.7	4.89	12.2	4.72
COP WEO Velocity (cm/s)	13.6	2.84	13.2	2.90	12.5	2.09	13.1	2.61
COP WEC Velocity (cm/s)	21.4	3.56	21.1	3.56	19.2	2.72	20.6	3.28
COP NEO Velocity (cm/s)	18.4	4.02	18.5	4.03	18.3	3.50	18.4	3.85
COP NEC Velocity (cm/s)	30.6	6.15	32.5	6.54	32.4	10.59	31.8	7.76
COP WEO Area (cm^2^)	14.2	2.90	14.3	3.13	16.1	5.69	14.9	3.91
COP WEC Area (cm^2^)	14.7	3.81	13.0	3.51	13.4	3.77	13.7	3.70
COP NEO Area (cm^2^)	18.0	5.85	18.9	6.77	17.4	7.70	18.1	6.77
COP NEC Area (cm^2^)	34.3	10.45	34.9	10.81	35.1	18.04	34.8	13.10
Speed (m/s)	0.9	0.22	1.0	0.23	0.8	0.13	0.9	0.19
Number of steps	18.5	7.82	20.5	3.11	19.2	5.55	19.2	5.50
Cadence (steps/min)	101.8	10.42	106.5	11.34	97.4	7.66	101.9	9.81
Step length—Left (cm)	50.2	11.02	56.5	9.66	47.5	6.89	51.4	9.19
Step length—Right (cm)	52.5	12.12	55.8	13.05	49.6	6.91	52.6	10.69
Step length—Difference (cm)	2.2	3.60	0.7	6.66	2.1	6.64	1.7	5.63
Step width—Left (cm)	10.5	3.93	8.2	3.77	8.5	3.94	9.1	3.88
Step width—Right (cm)	10.3	4.11	8.1	4.05	8.5	4.32	9.0	4.16
Step width—Difference (cm)	0.2	0.51	0.1	0.54	0.1	0.79	0.1	0.61
Total Gait Cycle—Left (%)	50.3	1.33	48.2	3.17	50.2	0.80	49.6	1.77
Total Gait Cycle—Right (%)	49.7	1.34	51.9	3.31	49.8	0.83	50.5	1.83
Swing phase—Left (%)	36.6	2.10	38.7	1.79	35.7	2.03	37.0	1.97
Swing phase—Right (%)	36.7	2.36	36.8	3.08	36.3	2.89	36.6	2.78
Stance phase—Left (%)	63.4	2.06	61.5	1.73	64.2	2.10	63.0	1.96
Stance phase—Right (%)	63.2	2.46	63.2	3.07	63.7	2.96	63.4	2.83
Double support phase—Left (%)	13.6	2.35	11.4	1.49	14.0	2.89	13.0	2.24
Double support phase—Right (%)	13.1	2.37	13.3	2.94	14.1	1.99	13.5	2.43

**Table 3:** Unadjusted and ANCOVA-adjusted effects of interventions on outcomes (*: nearly significant; **: significant; ***: highly significant changes).

	ROB		AG		EX	
Variable	Mean	SD	Mean	SD	Mean	SD
mRS (point)	−1.4 ***	0.50	−1.6 ***	0.52	−1.5 ***	0.69
BI (point)	11.8 ***	6.03	7.3 ***	6.67	12.7 ***	6.47
FIM (point)	22.2 ***	11.11	14.2 ***	6.55	19.4 ***	9.10
FIM-MS (point)	18.8 ***	8.40	18.6 ***	9.07	21.6 ***	7.69
SE-ADL (%)	8.2 **	8.74	7.3 ***	6.47	7.3 **	10.09
BBS (point)	7.4 ***	3.83	8.4 ***	3.91	11.9 ***	1.38
FES (point)	−22.8 ***	8.81	−23.6 ***	6.25	−20.8 ***	9.30
BESTest (point)	8.7 ***	9.05	8.8 ***	8.81	9.6 ***	6.77
6MWT (m)	128.2 ***	27.04	200.5 ***	60.31	182.7 ***	38.82
10mWT (m/s)	0.3 ***	0.19	0.4 ***	0.13	0.5 ***	0.14
COP WEO Sway (mm)	−6.4 ***	5.41	−11.0 ***	6.08	−9.1 ***	4.70
COP WEC Sway (mm)	−5.2 ***	4.86	0.2	0.42	0.7 *	1.26
COP NEO Sway (mm)	−2.8	5.36	−1.6	5.37	−2.6 **	3.07
COP NEC Sway (mm)	−6.2 ***	4.27	−3.1	7.42	−6.0 ***	5.36
COP WEO Velocity (cm/s)	−4.2 ***	1.90	−3.9 ***	1.55	−2.3 **	3.17
COP WEC Velocity (cm/s)	−6.7 ***	3.98	−6.1 ***	3.99	−2.7	5.77
COP NEO Velocity (cm/s)	−3.6 ***	3.68	−3.9 ***	3.24	−4.1 ***	3.86
COP NEC Velocity (cm/s)	−12.5 ***	4.71	−13.6 ***	6.62	−14.3 ***	8.87
COP WEO Area (cm^2^)	−4.1 ***	4.11	−4.2 ***	3.77	−3.9	7.65
COP WEC Area (cm^2^)	−2.6 *	4.40	−1.5 ***	4.04	−2.2	4.51
COP NEO Area (cm^2^)	−5.4 **	6.41	−5.9 ***	8.22	−4.0 *	6.51
COP NEC Area (cm^2^)	−14.6 ***	6.32	−14.8 ***	7.22	−15.4 ***	13.24

**Table 4:** Magnitude of the effects in the results of the posturography (Cohen’s d effect size).

Variable	ROB	AG	EX
COP WEO Sway	1.96	2.42	2.56
COP WEC Sway	1.51	0.22	0.73
COP NEO Sway	0.48	0.44	0.83
COP NEC Sway	2.44	0.60	1.50
COP WEO Velocity	1.86	1.63	0.97
COP WEC Velocity	2.21	2.04	0.77
COP NEO Velocity	1.00	1.11	1.28
COP NEC Velocity	2.66	2.59	1.81
COP WEO Area	1.26	1.27	0.86
COP WEC Area	0.77	0.53	0.66
COP NEO Area	1.09	1.02	0.63
COP NEC Area	1.59	1.67	1.14

**Table 5:** Effects of interventions on 3D motion capture outcomes (*: nearly significant; **: significant; ***: highly significant changes).

	ROB			AG			EX		
Variable	Mean	SD	Effect Size	Mean	SD	Effect Size	Mean	SD	Effect Size
Speed (m/s)	0.1	0.11	0.21	0.0	0.14	0.00	0.1 *	0.13	0.61
Cadence (steps/min)	0.7	7.34	0.08	−1.3	7.40	0.13	3.3	8.44	0.34
Number of steps	3.3 ***	2.87	0.52	−0.6	6.99	0.09	0.6	2.54	0.15
Step length—Left (cm)	1.4	5.54	0.11	−0.2	4.44	0.02	3.5 ***	5.09	0.52
Step length—Right (cm)	2.4 *	3.60	0.21	0.7	6.66	0.15	1.9	6.33	0.28
Step length—Diff. (cm)	−1.0	6.36	0.24	−1.9	4.80	0.30	1.6	3.81	0.26
Step width—Left (cm)	−2.5 **	2.63	0.76	−1.5	3.46	0.36	−1.0	4.14	0.25
Step width—Right (cm)	−2.3 **	2.81	0.68	−1.3	3.53	0.31	−0.7	4.12	0.17
Step width—Diff. (cm)	−0.2	0.64	0.36	−0.2	0.65	0.28	−0.3 *	0.50	0.43
Total Gait Cycle—Left (%)	−0.2	1.18	0.12	0.5	1.37	0.14	0.1	1.56	0.08
Total Gait Cycle—Right (%)	0.2	1.10	0.12	−0.4	1.25	0.14	−0.1	1.52	0.12
Swing phase—Left (%)	0.5	2.17	0.18	0.6	1.71	0.37	1.3 **	1.45	0.64
Swing phase—Right (%)	0.3	1.82	0.13	0.8 *	1.33	0.28	1.2	2.16	0.47
Stance phase—Left (%)	−0.5	2.14	0.18	−0.7	1.65	0.49	−1.2 ***	1.32	0.61
Stance phase—Right (%)	−0.2	1.77	0.08	−0.6	1.42	0.20	−1.1	2.23	0.45
Double support phase—Left (%)	−0.5	1.78	0.20	−0.3	1.52	0.24	−1.1 **	1.54	0.44
Double support phase—Right (%)	−0.3	2.36	0.09	−1.1 **	1.21	0.37	−1.4 **	2.04	0.68

## Data Availability

The data presented in this study is available on request from the corresponding author.

## Abbreviations

The following abbreviations are used in this manuscript:

VR	virtual reality
ROB	robotic training
AG	agility training
EX	exergaming
NIHSS	National Institutes of Health Stroke Scale
CT	computed tomography
MR	magnetic resonance imaging
BMI	Body Mass Index
mRS	modified Rankin Scale
RPE	rate of perceived exertion
BI	Barthel Index
FIM	Functional Independence Measure
FIM-MS	Functional Independence Measure-Motor Subscale
SE-ADL	The Schwab and England Activities of Daily Living Scale
BBS	Berg Balance Scale
BESTest	Balance Evaluation Systems Test
FES	Falls Efficacy Scale
WEO	wide stance-eye open
WEC	wide stance-eye closed
NEO	narrow stance-eye open
NEC	narrow stance-eye closed
COP	center of pressure
6mWT	6-min walk test
10mWT	10-m walk test
IMUs	inertial measurement units
3D	three-dimensional
